# Supplementary material for: Vitamin D Modified DSS-Induced Colitis in Mice via STING Signaling Pathway
Source: Biology (Basel). 2025 Jun 18;14(6):715. doi: 10.3390/biology14060715 (PMC12190092; doi:10.3390/biology14060715)
Supplement: Supplementary file 1 [file biology-14-00715-s001.zip › Supplementary Materials S1: 16s-report of gut microbiota/Groups/diff_analysis/RF/Order_Groups.RFimportance.pdf]

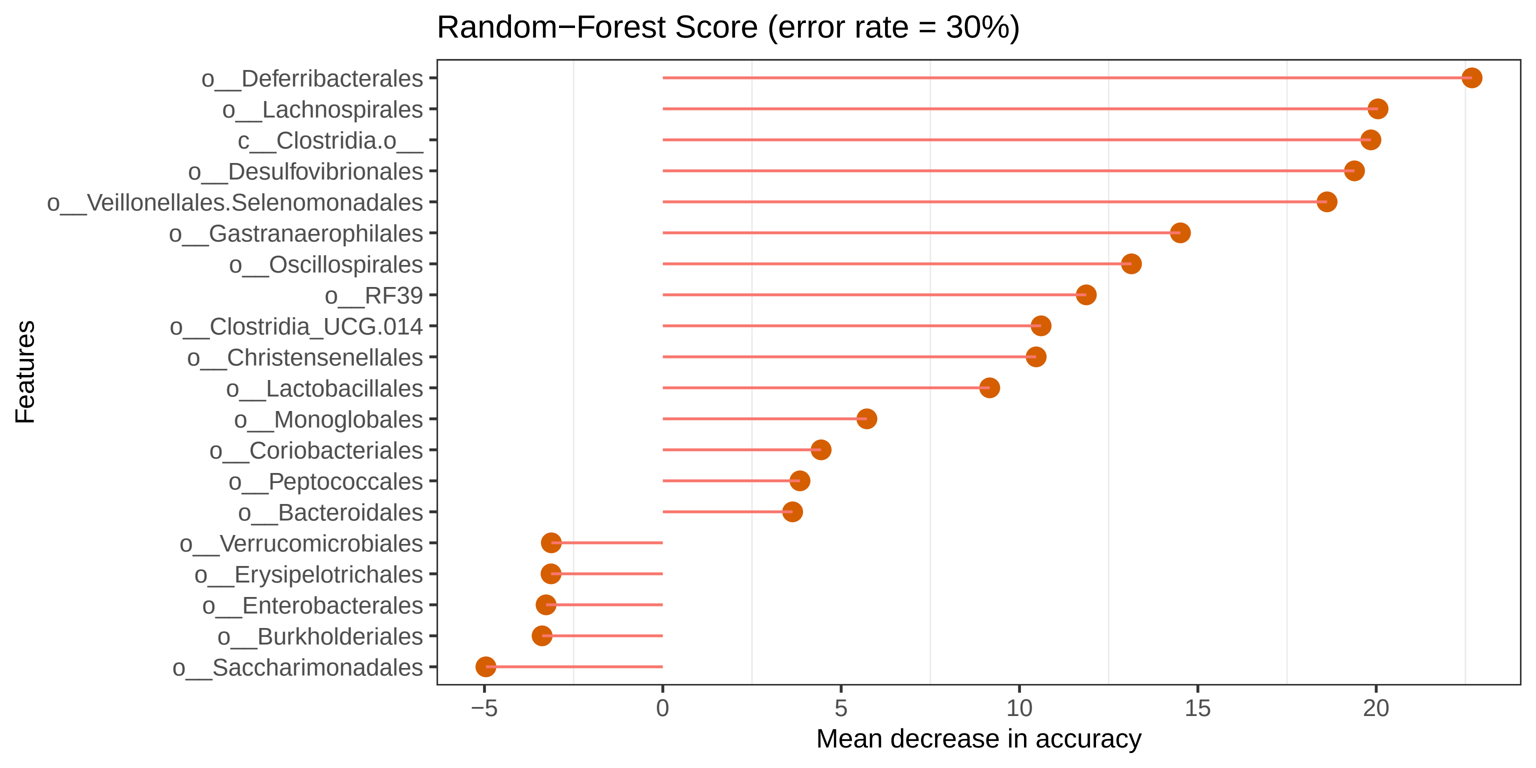
Random−Forest Score (error rate = 30%)
o__Deferribacterales
o__Lachnospirales
c__Clostridia.o__
o__Desulfovibrionales
o__Veillonellales.Selenomonadales
o__Gastranaerophilales
o__Oscillospirales
o__RF39
o__Clostridia_UCG.014
o__Christensenellales
o__Lactobacillales
o__Monoglobales
o__Coriobacteriales
o__Peptococcales
o__Bacteroidales
o__Verrucomicrobiales
o__Erysipelotrichales
o__Enterobacterales
o__Burkholderiales
o__Saccharimonadales
Features
−5
0
5
10
15
20
Mean decrease in accuracy
